# Supplementary material for: Preparations of trans- and cis-μ-1,2-Peroxodiiron(III) Complexes
Source: Molecules. 2023 Dec 29;29(1):205. doi: 10.3390/molecules29010205 (PMC10780643; doi:10.3390/molecules29010205)
Supplement: Supplementary file 1 [file molecules-29-00205-s001.zip › molecules-2785151-supplementary.pdf]

Supporting Information

Preparations of *trans-* and *cis*-μ-1,2-Peroxodiiron(III) Complexes

Yuji Kajita,[a] Masaki Kubo,[c] Hidekazu Arii,[b] Shinya Ishikawa,[c] Yamato Saito,[c] Yuko Wasada-Tsutsui,[c] Yasuhiro Funahashi,[d] Tomohiro Ozawa,[c] and Hideki Masuda[a,c]*

[a] Department of Applied Chemistry, Graduate School of Engineering, Aichi Institute of Techlology, Yakusa, Toyota 470-0392, Aichi, Japan

[b] Faculty of Education, Graduate School of Education, University of Miyazaki, 1-1 Gakuen Kibanadai-nishi, Miyazaki 889-2192, Miyazaki, Japan

[c] Department of Frontier Materials, Graduate School of Engineering, Nagoya Institute of Technology, Gokiso, Showa, Nagoya 466-8555, Aichi, Japan

[d] Department of Chemistry, Graduate School of Sciences, Osaka University, Machikaneyama, Toyonaka 560-0043, Osaka, Japan

Contents

Experimental

X-ray crystal structure analysis of complex **1**

Tables S1-S6. Crystallographic experimental details, final atomic coordinates, thermal parameters, full bond lengths and angles for **1**.

Table S7. Structural parameters, harmonic vibrational frequencies and Raman activities related to the O-O stretching mode for the small model.

Figure S1. Spectral change of the reaction of **1** and dioxygen in acetone at –60 ˚C.

Figure S2. Plots of $\ln(A_\infty/(A_\infty - A))$ vs t. for the reaction of **1** and dioxygen.

Figure S3. Resonance Raman spectrum of **2** ($^{16}O_2$) and calculated one separated by using Gaussian fitting program. The red and blue lines are observed and calculated spectra.

Figure S4. Raman active modes related to the O-O stretching in species **2**.

Figure S5. Raman active modes related to the O-O stretching in species **3**.

Experimental

X-ray crystal structure analysis of complex **1**

Data Collection

A colorless prism crystal of $C_{28}H_{35}Cl_4FeN_3$ having approximate dimensions of 0.50 x 0.40 x 0.40 mm was mounted on a glass fiber. All measurements were made on a Rigaku Mercury CCD area detector with graphite monochromated Mo-$K\alpha$ radiation. Indexing was performed from 0 images that were exposed for 0 seconds. The crystal-to-detector distance was 44.83 mm. Cell constants and an orientation matrix for data collection corresponded to a primitive triclinic cell with dimensions:

$a =$ 8.784(4) Å $\alpha =$ 71.432(11)^{o}

$b =$ 11.405(4) Å $\beta =$ 91.21(2)^{o}

$c =$ 16.653(8) Å $\gamma =$ 66.637(12)^{o}

$V =$ 1430.9(11) Å^3

For Z = 2 and F.W. = 611.26, the calculated density is 1.42 g/cm^3. Based on a statistical analysis of intensity distribution, and the successful solution and refinement of the structure, the space group was determined to be:

P-1 (#2)

The data were collected at a temperature of -100 $\pm$ 1°C to a maximum 2θ value of 55.0^{o}. A total of 1200 oscillation images were collected. A sweep of data was done using ω scans from -70.0 to 110.0^{o} in 0.3^{o} step, at χ=45.0^{o} and φ = 0.0^{o}. The exposure rate was 16.7 [sec./^{o}]. The detector swing angle was 20.17^{o}. A second sweep was performed using ω scans from -70.0 to 110.0^{o} in 0.3^{o} step, at χ=45.0^{o} and φ = 90.0^{o}. The exposure rate was 16.7 [sec./^{o}]. The detector swing angle was 20.17^{o}. The crystal-to-detector distance was 44.83 mm. Readout was performed in the 0.137 mm pixel mode.

Data Reduction

Of the 10888 reflections that were collected, 6134 were unique (R_{int} = 0.018); equivalent reflections were merged. Data were collected and processed using CrystalClear (Rigaku). Net intensities and sigmas were derived as follows:

$F^2 = [\Sigma(P_i - mB_{ave})] \cdot Lp^{-1}$

where P_i is the value in counts of the ith pixel

m is the number of pixels in the integration area

B_{ave} is the background average   Lp is the Lorentz and polarization factor

$B_{ave} = \Sigma(B_j)/n$

where n is the number of pixels in the background area

B_j is the value of the jth pixel in counts

$\sigma^2(F^2_{hkl}) = [(\Sigma P_i) + m((\Sigma(B_{ave} - B_j)^2)/(n-1))] \cdot Lp \cdot errmul + (erradd \cdot F^2)^2$

where erradd = 0.00   errmul = 1.00

The linear absorption coefficient, μ, for Mo-Kα radiation is 9.2 cm^{-1}. An empirical absorption correction was applied which resulted in transmission factors ranging from 0.89 to 1.00. The data were corrected for Lorentz and polarization effects.

Structure Solution and Refinement

The structure was solved by direct methods[2] and expanded using Fourier techniques[3]. The non-hydrogen atoms were refined

anisotropically. Hydrogen atoms were refined using the riding model. The final cycle of full-matrix least-squares refinement[4] on F^2 was based on 5705 observed reflections and 358 variable parameters and converged (largest parameter shift was 0.00 times its esd) with unweighted and weighted agreement factors of:

$$R1 = \Sigma ||Fo| - |Fc|| / \Sigma |Fo| = 0.039 \qquad wR2 = [\Sigma (w (Fo^2 - Fc^2)^2)/ \Sigma w(Fo^2)^2]^{1/2} = 0.080$$

The standard deviation of an observation of unit weight[5] was 0.95. A Sheldrick weighting scheme was used. Plots of Σ w ($|Fo| - |Fc|)^2$ versus |Fo|, reflection order in data collection, sin θ/λ and various classes of indices showed no unusual trends. The maximum and minimum peaks on the final difference Fourier map corresponded to 0.76 and -0.70 $e^-/Å^3$, respectively.

Neutral atom scattering factors were taken from Cromer and Waber[6]. Anomalous dispersion effects were included in Fcalc[7]; the values for Δf' and Δf" were those of Creagh and McAuley[8]. The values for the mass attenuation coefficients are those of Creagh and Hubbell[9]. All calculations were performed using the CrystalStructure[10,11] crystallographic software package.

References

(1) CrystalClear: Rigaku Corporation, 1999. CrystalClear Software User's Guide, Molecular Structure Corporation, (c) 2000.J.W.Pflugrath (1999) Acta Cryst. D55, 1718-1725.

(2) SAPI91: Fan Hai-Fu (1991). Structure Analysis Programs with Intelligent Control, Rigaku Corporation, Tokyo, Japan.

(3) DIRDIF99: Beurskens, P.T., Admiraal, G., Beurskens, G., Bosman, W.P., de Gelder, R., Israel, R. and Smits, J.M.M.(1999). The DIRDIF-99 program system, Technical Report of the Crystallography Laboratory, University of Nijmegen, The Netherlands.

(4) Least Squares function minimized:

$\Sigma w(F_o^2-F_c^2)^2$ where w = Least Squares weights.

(5) Standard deviation of an observation of unit weight:

$[\Sigma w(F_o^2-F_c^2)^2/(N_o-N_v)]^{1/2}$ where: N_o = number of observations N_v = number of variables

(6) Cromer, D. T. & Waber, J. T.; "International Tables for X-ray Crystallography", Vol. IV, The Kynoch Press, Birmingham, England, Table 2.2 A (1974).

(7) Ibers, J. A. & Hamilton, W. C.; Acta Crystallogr., 17, 781 (1964).

(8) Creagh, D. C. & McAuley, W.J .; "International Tables for Crystallography", Vol C, (A.J.C. Wilson, ed.), Kluwer Academic Publishers, Boston, Table 4.2.6.8, pages 219-222 (1992).

(9) Creagh, D. C. & Hubbell, J.H..; "International Tables for Crystallography", Vol C, (A.J.C. Wilson, ed.), Kluwer Academic Publishers, Boston, Table 4.2.4.3, pages 200-206 (1992).

(10) CrystalStructure 3.00: Crystal Structure Analysis Package, Rigaku and Rigaku/MSC (2000-2002).

(11) CRYSTALS Issue 10: Watkin, D.J., Prout, C.K. Carruthers, J.R. & Betteridge, P.W. Chemical Crystallography Laboratory, Oxford, UK.

Table S1. Crystallographic parameters for **1**

Formula		$C_{28}H_{35}Cl_4FeN_3$
Formula weight		611.26
Crystal system		triclinic
a / Å		8.784(4)
b / Å		11.405(4)
c / Å		16.653(8)
α / °		71.432(11)
β / °		91.21(2)
γ / °		66.637(12)°
V / Å^3		1430.9(11)
Space group		P-1
Z		2
D_{calc} / g cm^{-1}		1.419
F (000)		636.00
μ (Mo $K\alpha$) / cm^{-1}		9.22
T / K		173
λ (Mo $K\alpha$)		0.71070
$2\theta_{max}$ / deg		55.0
No. refls. measured	Total	10888
	Unique	6134 (R_{int} = 0.018)
No. observations ($I > 2.00\sigma$(I))		5705
No. variables		358
Residuals: $R1$ ($I > 2.00\sigma(I)$)		0.039
$wR2$ ($I > 2.00\sigma(I)$)		0.080
GOF		0.95
Max shift / error in final cycle		0.00
Maximum and Minimum peaks in final diff. map (e^-/Å^3)		0.76, -0.70

Table S2. Atomic coordinates and B_{iso}/B_{eq}

atom	x	y	z	B_{eq}
Fe(1)	0.94266(3)	0.01112(3)	0.83162(2)	1.344(5)
Cl(1)	1.08333(6)	0.09387(5)	0.72220(3)	2.320(10)
Cl(2)	1.17700(6)	-0.08767(5)	0.94295(3)	2.133(9)
Cl(3)	0.80705(12)	0.52000(8)	0.43384(5)	5.50(2)
Cl(4)	0.29963(11)	0.68857(9)	0.47305(5)	5.24(2)
N(1)	0.8247(2)	-0.1007(2)	0.92519(10)	1.48(3)
N(2)	0.8001(2)	-0.0080(2)	0.73058(10)	1.64(3)
N(3)	0.7237(2)	0.1941(2)	0.82738(10)	1.51(3)
C(1)	0.6416(2)	-0.0612(2)	0.90095(12)	1.69(4)
C(2)	0.6023(2)	-0.0807(2)	0.81758(13)	1.89(4)
C(3)	0.6210(2)	0.0194(2)	0.73682(12)	1.77(4)
C(4)	0.5218(2)	0.1665(2)	0.73289(12)	1.78(4)
C(5)	0.5556(2)	0.1919(2)	0.81508(12)	1.56(3)
C(6)	0.5382(2)	0.0866(2)	0.89319(12)	1.73(4)
C(7)	0.9235(3)	-0.2516(2)	0.95886(12)	1.85(4)
C(8)	0.8877(2)	-0.3261(2)	1.04395(12)	1.76(4)
C(9)	0.8381(3)	-0.4304(2)	1.0523(2)	2.29(4)
C(10)	0.8190(3)	-0.5061(2)	1.1323(2)	2.82(5)
C(11)	0.8477(3)	-0.4779(2)	1.2040(2)	2.67(4)
C(12)	0.8916(3)	-0.3716(2)	1.1963(1)	2.47(4)
C(13)	0.9110(3)	-0.2960(2)	1.11644(13)	2.11(4)
C(14)	0.8983(3)	-0.1373(2)	0.71514(13)	2.08(4)
C(15)	0.8286(3)	-0.1448(2)	0.63410(12)	1.95(4)
C(16)	0.8358(4)	-0.0615(3)	0.5546(2)	3.65(6)
C(17)	0.7697(5)	-0.0668(3)	0.4797(2)	4.82(7)
C(18)	0.7002(4)	-0.1564(3)	0.4843(2)	4.14(6)
C(19)	0.6931(4)	-0.2395(3)	0.5626(2)	3.99(7)
C(20)	0.7564(3)	-0.2335(3)	0.6375(2)	2.91(5)
C(21)	0.7267(3)	0.3212(2)	0.76706(12)	1.92(4)
C(22)	0.6111(3)	0.4534(2)	0.7772(1)	2.17(4)
C(23)	0.6234(3)	0.4763(3)	0.8532(2)	3.32(5)
C(24)	0.5217(3)	0.6029(3)	0.8581(2)	4.54(7)
C(25)	0.4091(3)	0.7054(3)	0.7870(3)	4.66(7)
C(26)	0.3952(3)	0.6837(3)	0.7119(2)	4.32(6)
C(27)	0.4950(3)	0.5589(2)	0.7067(2)	3.05(5)

Table S2. Atomic coordinates and B_{iso}/B_{eq} (continued)

atom	x	y	z	B_{eq}
C(28)	0.1367(4)	0.6353(4)	0.4828(3)	7.00(9)
H(1)	0.8308(2)	-0.0754(2)	0.97385(10)	1.69(8)
H(2)	0.7952(2)	0.0621(2)	0.67952(10)	1.95(9)
H(3)	0.7364(2)	0.1996(2)	0.88270(10)	1.73(8)
H(4)	0.6097(2)	-0.1181(2)	0.94582(12)	1.95(9)
H(5)	0.6765(2)	-0.1707(2)	0.82209(13)	2.31(9)
H(6)	0.4895(2)	-0.0707(2)	0.81177(13)	2.31(9)
H(7)	0.5742(2)	0.0101(2)	0.68851(12)	2.09(9)
H(8)	0.5501(2)	0.2250(2)	0.68717(12)	1.94(9)
H(9)	0.4053(2)	0.1891(2)	0.72137(12)	1.94(9)
H(10)	0.4721(2)	0.2793(2)	0.81015(12)	1.73(9)
H(11)	0.4230(2)	0.1029(2)	0.88989(12)	2.04(9)
H(12)	0.5736(2)	0.0981(2)	0.94331(12)	2.05(9)
H(13)	0.8992(3)	-0.2858(2)	0.91755(12)	2.03(9)
H(14)	1.0396(3)	-0.2707(2)	0.96568(12)	2.04(9)
H(15)	0.9402(3)	-0.2225(2)	1.11114(13)	2.37(9)
H(16)	0.9087(3)	-0.3502(2)	1.2455(1)	2.69(9)
H(17)	0.8377(3)	-0.5316(2)	1.2587(2)	2.70(9)
H(18)	0.7855(3)	-0.5775(2)	1.1374(2)	3.25(10)
H(19)	0.8176(3)	-0.4503(2)	1.0030(2)	2.71(9)
H(20)	1.0098(3)	-0.1459(2)	0.71161(13)	2.40(9)
H(21)	0.9003(3)	-0.2113(2)	0.76270(13)	2.41(9)
H(22)	0.8854(4)	0.0001(3)	0.5510(2)	4.71(11)
H(23)	0.7727(5)	-0.0076(3)	0.4253(2)	5.18(11)
H(24)	0.6572(4)	-0.1602(3)	0.4328(2)	4.62(11)
H(25)	0.6457(4)	-0.3023(3)	0.5658(2)	5.53(12)
H(26)	0.7495(3)	-0.2903(3)	0.6919(2)	3.92(10)
H(27)	0.8385(3)	0.3130(2)	0.77451(12)	2.16(9)
H(28)	0.6959(3)	0.3275(2)	0.71030(12)	2.15(9)
H(29)	0.7011(3)	0.4054(3)	0.9022(2)	3.98(10)
H(30)	0.5303(3)	0.6183(3)	0.9105(2)	6.27(13)
H(31)	0.3404(3)	0.7915(3)	0.7907(3)	5.84(13)
H(32)	0.3173(3)	0.7549(3)	0.6631(2)	4.50(11)
H(33)	0.4849(3)	0.5438(2)	0.6543(2)	3.23(10)

$B_{eq} = 8/3\ \pi^2(U_{11}(aa^*)^2 + U_{22}(bb^*)^2 + U_{33}(cc^*)^2 + 2U_{12}(aa^*bb^*)\cos\gamma + 2U_{13}(aa^*cc^*)\cos\beta + 2U_{23}(bb^*cc^*)\cos\alpha)$

Table S3. Anisotropic Displacement Parameters

atom	U_{11}	U_{22}	U_{33}	U_{12}	U_{13}	U_{23}
Fe(1)	0.01595(13)	0.01693(13)	0.01540(13)	-0.00450(10)	0.00127(9)	-0.00553(10)
Cl(1)	0.0273(2)	0.0352(3)	0.0247(2)	-0.0142(2)	0.0094(2)	-0.0082(2)
Cl(2)	0.0229(2)	0.0308(3)	0.0230(2)	-0.0085(2)	-0.0029(2)	-0.0084(2)
Cl(3)	0.0906(6)	0.0541(4)	0.0540(4)	-0.0372(4)	-0.0088(4)	0.0006(4)
Cl(4)	0.0657(5)	0.0775(5)	0.0415(4)	-0.0385(4)	-0.0046(3)	0.0067(4)
N(1)	0.0193(7)	0.0160(7)	0.0179(7)	-0.0058(6)	0.0016(6)	-0.0043(6)
N(2)	0.0204(8)	0.0227(8)	0.0182(7)	-0.0065(6)	0.0027(6)	-0.0093(6)
N(3)	0.0183(7)	0.0163(7)	0.0189(7)	-0.0049(6)	0.0009(6)	-0.0050(6)
C(1)	0.0197(9)	0.0199(9)	0.0226(9)	-0.0086(7)	0.0048(7)	-0.0050(7)
C(2)	0.0207(9)	0.0224(9)	0.0300(10)	-0.0094(8)	0.0018(8)	-0.0106(8)
C(3)	0.0184(9)	0.0259(10)	0.0217(9)	-0.0069(8)	-0.0019(7)	-0.0103(8)
C(4)	0.0186(9)	0.0217(9)	0.0208(9)	-0.0043(7)	-0.0018(7)	-0.0049(7)
C(5)	0.0154(8)	0.0173(9)	0.0226(9)	-0.0024(7)	0.0020(7)	-0.0077(7)
C(6)	0.0183(9)	0.0246(9)	0.0215(9)	-0.0073(8)	0.0052(7)	-0.0086(7)
C(7)	0.0239(9)	0.0158(8)	0.0249(9)	-0.0048(7)	0.0031(7)	-0.0049(7)
C(8)	0.0173(9)	0.0159(9)	0.0260(10)	-0.0036(7)	0.0011(7)	-0.0024(7)
C(9)	0.0247(10)	0.0218(10)	0.0390(12)	-0.0090(8)	0.0053(9)	-0.0102(9)
C(10)	0.0275(11)	0.0220(10)	0.053(1)	-0.0122(9)	0.0107(10)	-0.0055(10)
C(11)	0.0230(10)	0.0265(11)	0.0357(12)	-0.0053(9)	0.0098(9)	0.0029(9)
C(12)	0.0262(10)	0.0319(11)	0.0263(10)	-0.0086(9)	0.0029(8)	-0.0035(9)
C(13)	0.0262(10)	0.0220(9)	0.0268(10)	-0.0102(8)	0.0005(8)	-0.0027(8)
C(14)	0.0232(9)	0.0285(10)	0.0246(10)	-0.0045(8)	0.0024(8)	-0.0146(8)
C(15)	0.0245(10)	0.0265(10)	0.0218(9)	-0.0059(8)	0.0053(7)	-0.0134(8)
C(16)	0.082(2)	0.0369(13)	0.0301(12)	-0.0309(13)	0.0200(12)	-0.0173(10)
C(17)	0.104(3)	0.038(1)	0.0211(12)	-0.013(2)	0.009(1)	-0.0090(11)
C(18)	0.051(2)	0.058(2)	0.037(1)	-0.0025(13)	-0.0101(12)	-0.0303(13)
C(19)	0.050(2)	0.071(2)	0.054(2)	-0.033(1)	0.0114(13)	-0.041(2)
C(20)	0.0457(13)	0.047(1)	0.0309(11)	-0.0263(11)	0.0131(10)	-0.0215(10)
C(21)	0.0254(10)	0.0186(9)	0.0243(10)	-0.0082(8)	0.0007(8)	-0.0037(7)
C(22)	0.0209(9)	0.0187(9)	0.0416(12)	-0.0098(8)	0.0006(8)	-0.0078(8)
C(23)	0.0264(11)	0.0392(13)	0.061(2)	-0.0053(10)	-0.0041(11)	-0.0301(12)
C(24)	0.0334(13)	0.059(2)	0.106(3)	-0.0220(13)	0.013(2)	-0.059(2)
C(25)	0.0263(12)	0.0236(12)	0.135(3)	-0.0143(10)	0.018(2)	-0.033(2)
C(26)	0.0332(13)	0.0194(11)	0.089(2)	-0.0070(10)	0.010(1)	0.0035(13)

Table S3. Anisotropic Displacement Parameters (continued)

atom	U_{11}	U_{22}	U_{33}	U_{12}	U_{13}	U_{23}
C(27)	0.0274(11)	0.0244(11)	0.050(1)	-0.0092(9)	0.0033(10)	0.0025(10)
C(28)	0.051(2)	0.068(2)	0.095(3)	-0.028(2)	-0.016(2)	0.037(2)

The general temperature factor expression: $\exp(-2\pi^2(a^{*2}U_{11}h^2 + b^{*2}U_{22}k^2 + c^{*2}U_{33}l^2 + 2a^*b^*U_{12}hk + 2a^*c^*U_{13}hl + 2b^*c^*U_{23}kl))$

Table S4. Bond lengths (Å)

atom	atom	distance	atom	atom	distance
Fe(1)	Cl(1)	2.3800(10)	Fe(1)	Cl(2)	2.3690(10)
Fe(1)	N(1)	2.231(2)	Fe(1)	N(2)	2.193(2)
Fe(1)	N(3)	2.186(2)	Cl(4)	C(28)	1.756(4)
N(1)	C(1)	1.495(2)	N(1)	C(7)	1.486(2)
N(2)	C(3)	1.491(2)	N(2)	C(14)	1.492(2)
N(3)	C(5)	1.498(2)	N(3)	C(21)	1.489(2)
C(1)	C(2)	1.528(3)	C(1)	C(6)	1.525(3)
C(2)	C(3)	1.523(3)	C(3)	C(4)	1.532(3)
C(4)	C(5)	1.533(3)	C(5)	C(6)	1.523(3)
C(7)	C(8)	1.514(3)	C(8)	C(9)	1.391(3)
C(8)	C(13)	1.387(3)	C(9)	C(10)	1.394(3)
C(10)	C(11)	1.374(4)	C(11)	C(12)	1.382(3)
C(12)	C(13)	1.392(3)	C(14)	C(15)	1.511(3)
C(15)	C(16)	1.382(3)	C(15)	C(20)	1.379(3)
C(16)	C(17)	1.394(4)	C(17)	C(18)	1.368(5)
C(18)	C(19)	1.366(4)	C(19)	C(20)	1.388(3)
C(21)	C(22)	1.512(3)	C(22)	C(23)	1.382(3)
C(22)	C(27)	1.394(3)	C(23)	C(24)	1.395(4)
C(24)	C(25)	1.379(5)	C(25)	C(26)	1.361(5)
C(26)	C(27)	1.377(4)			

Table S5. Bond angles (°)

atom	atom	atom	angle	atom	atom	atom	angle
Cl(1)	Fe(1)	Cl(2)	94.46(4)	Cl(1)	Fe(1)	N(1)	168.78(4)
Cl(2)	Fe(1)	N(1)	87.00(6)	Cl(1)	Fe(1)	N(2)	86.12(6)
Cl(2)	Fe(1)	N(2)	149.43(5)	N(1)	Fe(1)	N(2)	87.00(6)
Cl(1)	Fe(1)	N(3)	104.24(5)	Cl(2)	Fe(1)	N(3)	119.40(5)
N(1)	Fe(1)	N(3)	84.57(6)	N(2)	Fe(1)	N(3)	89.78(7)
Fe(1)	N(1)	C(1)	117.17(11)	Fe(1)	N(1)	C(7)	113.82(12)
C(1)	N(1)	C(7)	111.6(1)	Fe(1)	N(2)	C(3)	116.93(11)
Fe(1)	N(2)	C(14)	110.82(12)	C(3)	N(2)	C(14)	114.0(2)
Fe(1)	N(3)	C(5)	116.35(11)	Fe(1)	N(3)	C(21)	112.05(12)
C(5)	N(3)	C(21)	112.0(1)	N(1)	C(1)	C(2)	112.5(2)
N(1)	C(1)	C(6)	110.2(2)	C(2)	C(1)	C(6)	110.0(2)
C(1)	C(2)	C(3)	114.7(2)	N(2)	C(3)	C(2)	112.6(2)
N(2)	C(3)	C(4)	110.0(2)	C(2)	C(3)	C(4)	110.3(2)
C(3)	C(4)	C(5)	114.8(2)	N(3)	C(5)	C(4)	112.7(2)
N(3)	C(5)	C(6)	110.6(2)	C(4)	C(5)	C(6)	110.6(2)
C(1)	C(6)	C(5)	114.2(2)	N(1)	C(7)	C(8)	115.1(2)
C(7)	C(8)	C(9)	120.8(2)	C(7)	C(8)	C(13)	120.7(2)
C(9)	C(8)	C(13)	118.4(2)	C(8)	C(9)	C(10)	120.5(2)
C(9)	C(10)	C(11)	120.4(2)	C(10)	C(11)	C(12)	119.8(2)
C(11)	C(12)	C(13)	119.8(2)	C(8)	C(13)	C(12)	121.0(2)
N(2)	C(14)	C(15)	114.5(2)	C(14)	C(15)	C(16)	120.0(2)
C(14)	C(15)	C(20)	121.3(2)	C(16)	C(15)	C(20)	118.7(2)
C(15)	C(16)	C(17)	120.3(3)	C(16)	C(17)	C(18)	120.3(3)
C(17)	C(18)	C(19)	119.9(2)	C(18)	C(19)	C(20)	120.3(3)
C(15)	C(20)	C(19)	120.7(2)	N(3)	C(21)	C(22)	116.2(2)
C(21)	C(22)	C(23)	121.9(2)	C(21)	C(22)	C(27)	119.5(2)
C(23)	C(22)	C(27)	118.5(2)	C(22)	C(23)	C(24)	120.1(3)
C(23)	C(24)	C(25)	119.9(3)	C(24)	C(25)	C(26)	120.5(2)
C(25)	C(26)	C(27)	119.8(3)	C(22)	C(27)	C(26)	121.1(3)

Table S6. Torsion Angles (°)

atom	atom	atom	atom	angle	atom	atom	atom	atom	angle
Cl(1)	Fe(1)	N(1)	C(1)	-94.6(6)	Cl(1)	Fe(1)	N(1)	C(7)	38.1(7)
Cl(2)	Fe(1)	N(1)	C(1)	167.6(3)	Cl(2)	Fe(1)	N(1)	C(7)	-59.7(2)
N(2)	Fe(1)	N(1)	C(1)	-42.4(3)	N(2)	Fe(1)	N(1)	C(7)	90.3(3)
N(3)	Fe(1)	N(1)	C(1)	47.7(3)	N(3)	Fe(1)	N(1)	C(7)	-179.6(3)
Cl(1)	Fe(1)	N(2)	C(3)	-145.4(3)	Cl(1)	Fe(1)	N(2)	C(14)	81.7(2)
Cl(2)	Fe(1)	N(2)	C(3)	122.4(3)	Cl(2)	Fe(1)	N(2)	C(14)	-10.5(4)
N(1)	Fe(1)	N(2)	C(3)	43.5(3)	N(1)	Fe(1)	N(2)	C(14)	-89.4(3)
N(3)	Fe(1)	N(2)	C(3)	-41.1(3)	N(3)	Fe(1)	N(2)	C(14)	-174.0(3)
Cl(1)	Fe(1)	N(3)	C(5)	124.4(2)	Cl(1)	Fe(1)	N(3)	C(21)	-6.3(3)
Cl(2)	Fe(1)	N(3)	C(5)	-132.0(2)	Cl(2)	Fe(1)	N(3)	C(21)	97.3(2)
N(1)	Fe(1)	N(3)	C(5)	-48.5(3)	N(1)	Fe(1)	N(3)	C(21)	-179.3(3)
N(2)	Fe(1)	N(3)	C(5)	38.5(3)	N(2)	Fe(1)	N(3)	C(21)	-92.3(3)
Fe(1)	N(1)	C(1)	C(2)	59.6(3)	Fe(1)	N(1)	C(1)	C(6)	-63.6(2)
C(7)	N(1)	C(1)	C(2)	-74.1(3)	C(7)	N(1)	C(1)	C(6)	162.7(3)
Fe(1)	N(1)	C(7)	C(8)	157.8(2)	C(1)	N(1)	C(7)	C(8)	-66.9(3)
Fe(1)	N(2)	C(3)	C(2)	-62.7(3)	Fe(1)	N(2)	C(3)	C(4)	60.8(2)
C(14)	N(2)	C(3)	C(2)	68.8(4)	C(14)	N(2)	C(3)	C(4)	-167.7(3)
Fe(1)	N(2)	C(14)	C(15)	-166.9(2)	C(3)	N(2)	C(14)	C(15)	58.7(4)
Fe(1)	N(3)	C(5)	C(4)	-57.4(2)	Fe(1)	N(3)	C(5)	C(6)	67.0(2)
C(21)	N(3)	C(5)	C(4)	73.4(3)	C(21)	N(3)	C(5)	C(6)	-162.3(3)
Fe(1)	N(3)	C(21)	C(22)	-163.3(2)	C(5)	N(3)	C(21)	C(22)	63.8(3)
N(1)	C(1)	C(2)	C(3)	-70.0(3)	C(6)	C(1)	C(2)	C(3)	53.3(4)
N(1)	C(1)	C(6)	C(5)	71.3(3)	C(2)	C(1)	C(6)	C(5)	-53.4(4)
C(1)	C(2)	C(3)	N(2)	71.7(3)	C(1)	C(2)	C(3)	C(4)	-51.7(3)
N(2)	C(3)	C(4)	C(5)	-74.6(3)	C(2)	C(3)	C(4)	C(5)	50.2(4)
C(3)	C(4)	C(5)	N(3)	73.7(3)	C(3)	C(4)	C(5)	C(6)	-50.7(3)
N(3)	C(5)	C(6)	C(1)	-73.3(3)	C(4)	C(5)	C(6)	C(1)	52.2(3)
N(1)	C(7)	C(8)	C(9)	124.1(3)	N(1)	C(7)	C(8)	C(13)	-58.9(4)
C(7)	C(8)	C(9)	C(10)	174.6(3)	C(13)	C(8)	C(9)	C(10)	-2.5(5)
C(7)	C(8)	C(13)	C(12)	-174.7(3)	C(9)	C(8)	C(13)	C(12)	2.4(5)
C(8)	C(9)	C(10)	C(11)	0.4(5)	C(9)	C(10)	C(11)	C(12)	1.7(5)
C(10)	C(11)	C(12)	C(13)	-1.8(5)	C(11)	C(12)	C(13)	C(8)	-0.3(5)
N(2)	C(14)	C(15)	C(16)	69.2(4)	N(2)	C(14)	C(15)	C(20)	-110.6(3)
C(14)	C(15)	C(16)	C(17)	-179.2(3)	C(20)	C(15)	C(16)	C(17)	0.6(6)

Table S6. Torsion Angles(°) (continued)

atom	atom	atom	atom	angle	atom	atom	atom	atom	angle
C(14)	C(15)	C(20)	C(19)	-179.9(3)	C(16)	C(15)	C(20)	C(19)	0.4(6)
C(15)	C(16)	C(17)	C(18)	-1.3(6)	C(16)	C(17)	C(18)	C(19)	1.1(6)
C(17)	C(18)	C(19)	C(20)	-0.1(7)	C(18)	C(19)	C(20)	C(15)	-0.6(6)
N(3)	C(21)	C(22)	C(23)	59.1(4)	N(3)	C(21)	C(22)	C(27)	-124.8(3)
C(21)	C(22)	C(23)	C(24)	175.7(3)	C(27)	C(22)	C(23)	C(24)	-0.3(6)
C(21)	C(22)	C(27)	C(26)	-175.8(3)	C(23)	C(22)	C(27)	C(26)	0.4(6)
C(22)	C(23)	C(24)	C(25)	-0.1(6)	C(23)	C(24)	C(25)	C(26)	0.5(6)
C(24)	C(25)	C(26)	C(27)	-0.5(7)	C(25)	C(26)	C(27)	C(22)	-0.0(6)

Table S7. Structural parameters, harmonic vibrational frequencies and Raman activities related to the O-O stretching mode for the small model.

	Small model of **2**		Small model of **3**	
	dichlorido	tetrachlorido	monoformato[a]	diformato
$S = 0$				
O-O (Å)	1.335	1.381	1.475	1.360
Fe-Fe (Å)	4.258	4.362	3.554	3.835
Fe-O-O-Fe (deg.)	–82.0	–126.5	135.9	–58.9
ν(O-O) (cm^{-1})	939, 947	938	820, 859	927
Raman activ.[b]	2.5, 2.6(×10^3)	1.6 (×10^4)	1.5, 0.4 (×10^2)	4.0×10^3
$S = 5$				
O-O (Å)	1.369	1.395	1.469	1.386
Fe-Fe (Å)	4.234	4.371	3.585	3.793
Fe-O-O-Fe (deg.)	–87.6	–129.4	132.6	–61.2
ν(O-O) (cm^{-1})	914	927	818	901
Raman activ.[b]	5.0×10^3	1.3 ×10^4	3.2×10^2	3.9×10^3

[a]O_2 binding complex with side-on mode.

[b]Raman activity. The unit is KM/mol. The modes denoted in Table are to partly contain the O-O stretching mode having Raman activities down to the one-fourth of the largest one.

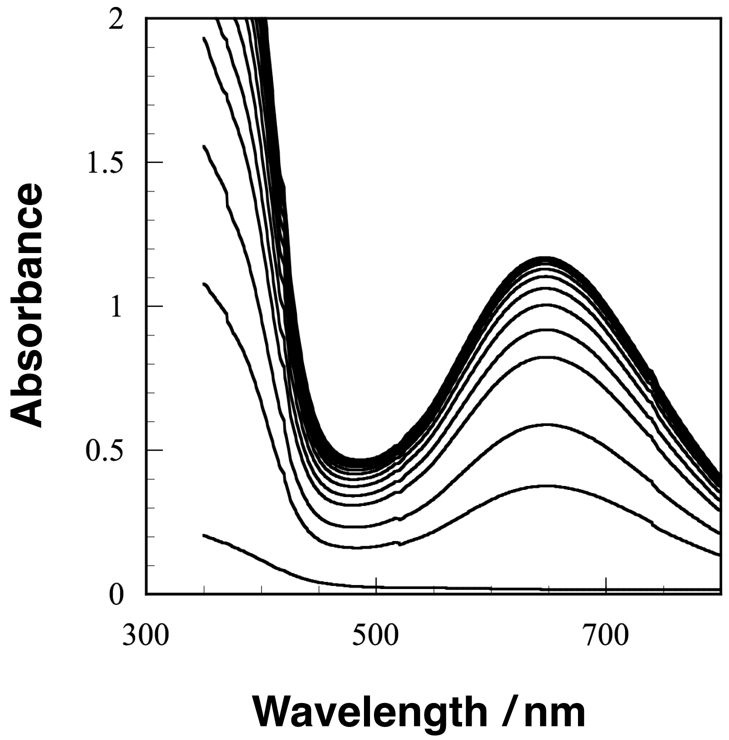

Figure S1. Spectral change of the reaction of **1** and dioxygen in acetone at –60 ˚C.

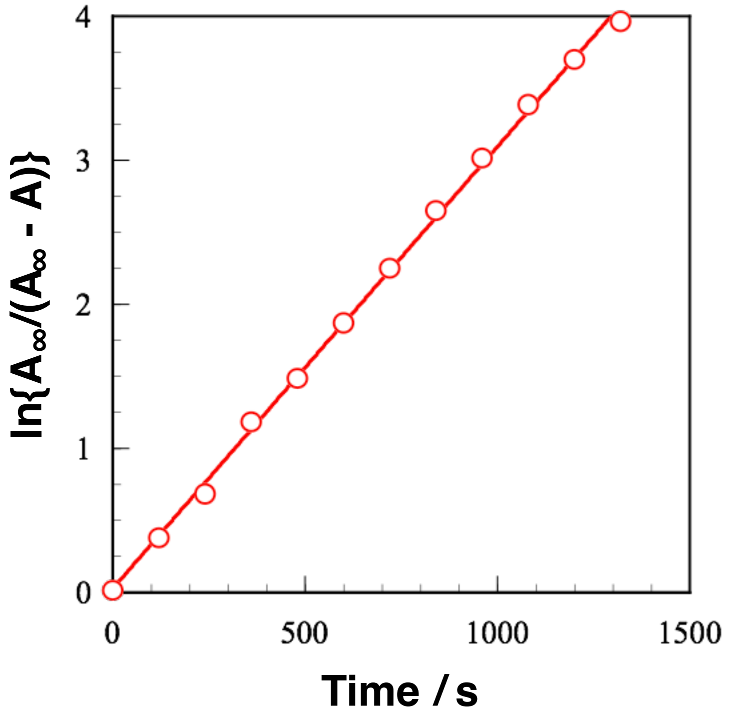

Figure S2. Plots of $\ln(A_\infty/(A_\infty - A))$ vs t for the reaction of **1** and dioxygen in acetone at –60 °C.

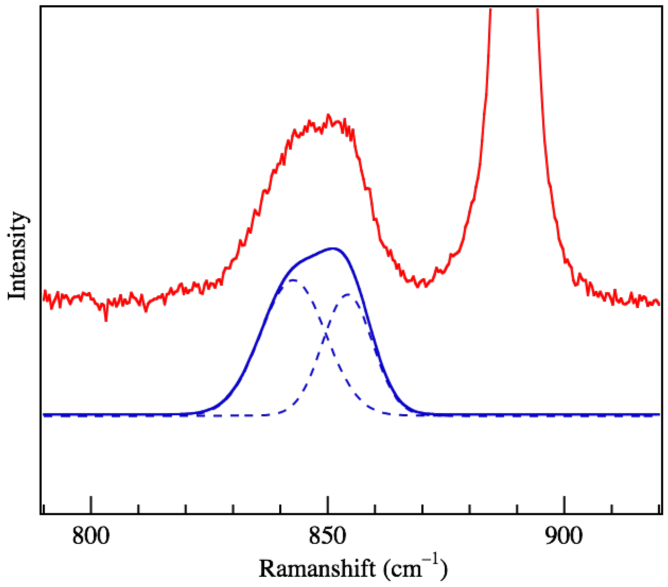

Figure S3. Resonance Raman spectrum of **2** ($^{16}O_2$) and calculated one separated by using Gaussian fitting program. The red and blue lines are observed and calculated spectra.

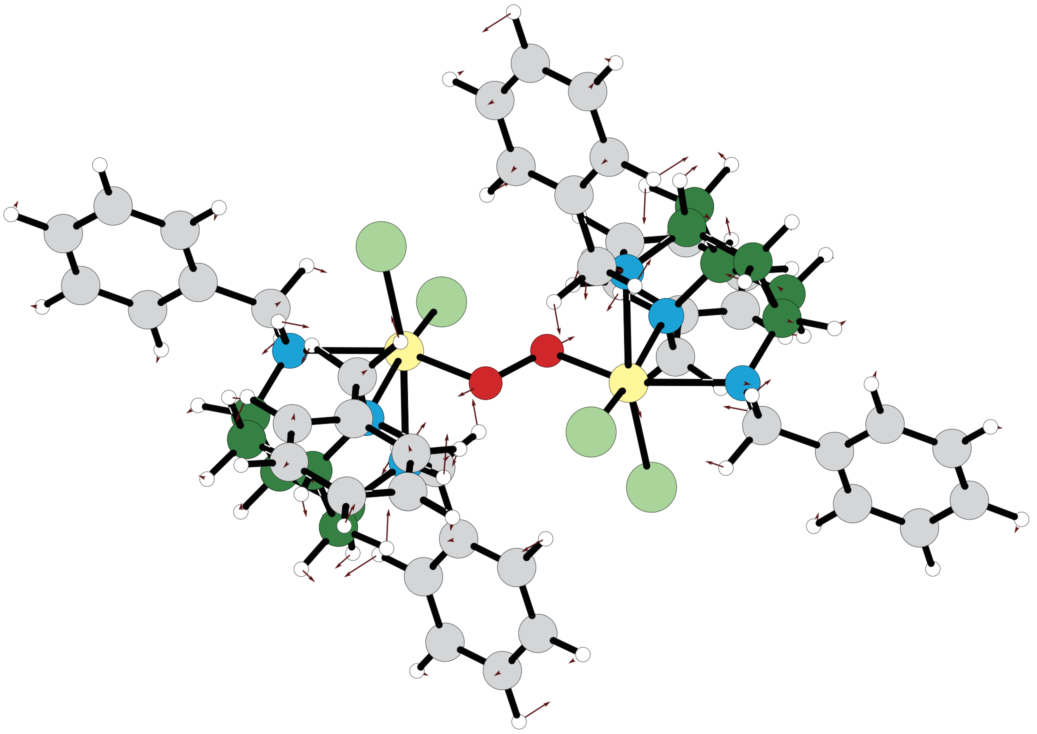

164: 917 cm⁻¹

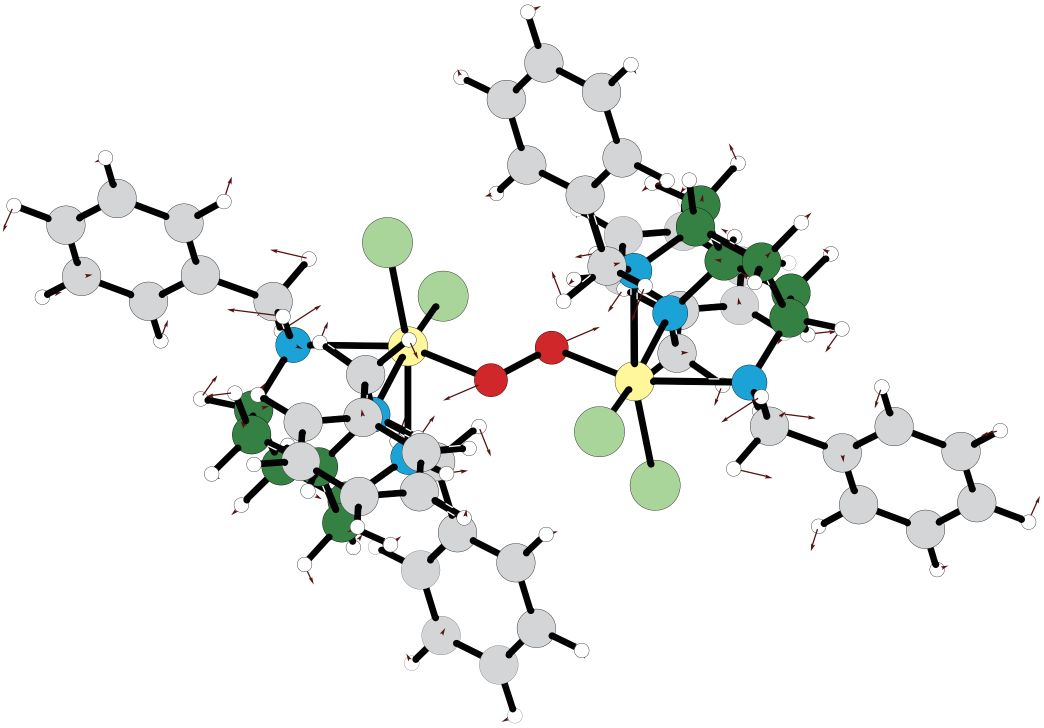

166: 926 cm⁻¹

Figure S4. Raman active modes related to the O-O stretching in species **2**.

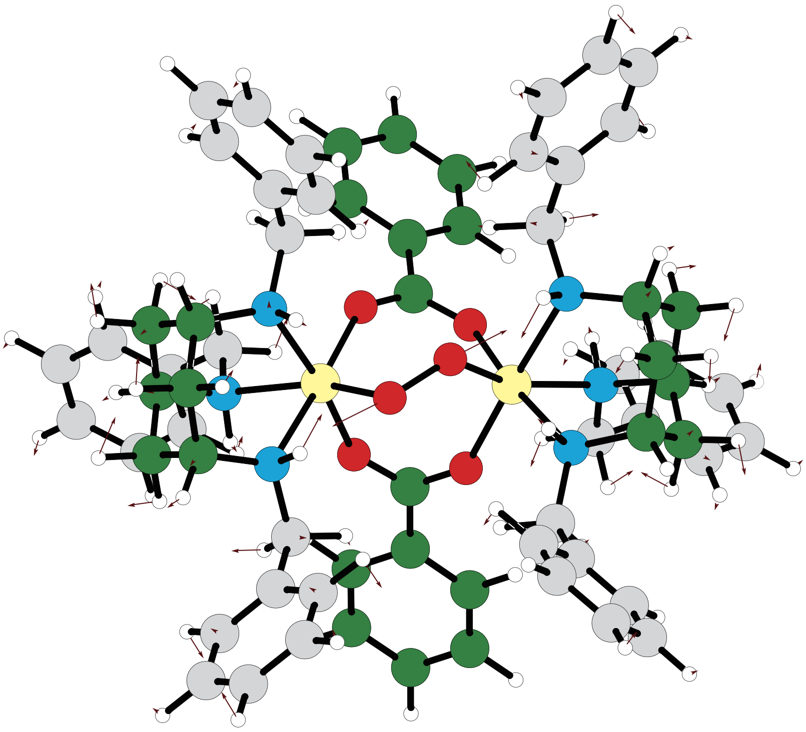

Figure S5. Raman active modes related to the O-O stretching in species **3**.
